# Jasmonates and Plant Salt Stress: Molecular Players, Physiological Effects, and Improving Tolerance by Using Genome-Associated Tools

**DOI:** 10.3390/ijms22063082

**Published:** 2021-03-17

**Authors:** Celia Delgado, Freddy Mora-Poblete, Sunny Ahmar, Jen-Tsung Chen, Carlos R. Figueroa

**Affiliations:** 1 Institute of Biological Sciences, Campus Talca, Universidad de Talca, Talca 3465548, Chile; celia.delgado@utalca.cl (C.D.); fmora@utalca.cl (F.M.-P.); sunny.ahmar@utalca.cl (S.A.); 2 Department of Life Sciences, College of Science, National University of Kaohsiung, Kaohsiung 811, Taiwan; jentsung@nuk.edu.tw

**Keywords:** salt stress, jasmonates, jasmonate signaling pathway, crosstalk, exogenous jasmonate applications, GWAS

## Abstract

Soil salinity is one of the most limiting stresses for crop productivity and quality worldwide. In this sense, jasmonates (JAs) have emerged as phytohormones that play essential roles in mediating plant response to abiotic stresses, including salt stress. Here, we reviewed the mechanisms underlying the activation and response of the JA-biosynthesis and JA-signaling pathways under saline conditions in *Arabidopsis* and several crops. In this sense, molecular components of JA-signaling such as MYC2 transcription factor and JASMONATE ZIM-DOMAIN (JAZ) repressors are key players for the JA-associated response. Moreover, we review the antagonist and synergistic effects between JA and other hormones such as abscisic acid (ABA). From an applied point of view, several reports have shown that exogenous JA applications increase the antioxidant response in plants to alleviate salt stress. Finally, we discuss the latest advances in genomic techniques for the improvement of crop tolerance to salt stress with a focus on jasmonates.

## 1. Introduction

Salinity is a serious hazard for agriculture since most of the crop plants are salt-sensitive [1]. Current data show that global soil salinization increased by more than 100 Mha between 1986 and 2016 and it is expanding on a global scale, approximately at a rate of 2 Mha per year [2]. Thus, the future of food supplies for animals and humankind is threatened [3]. Natural processes such as geological deposits due to parent rock constituents, salinized groundwater, marine transgressions, storm flood events, tsunamis, and recurrent drought events and the general increase in temperature [4,5] cause soil salinization. Unfortunately, human interventions have also promoted the increment of saline lands. Wrong irrigation practices, poor drainage conditions [6], the use of fertilizers [7], mismanagement of treated wastewater [8], industrial [9], and mining operation effluents enriched with a salt contribute to the salinity increment in the soil and water [10].

Under salt stress conditions, physiological and metabolic activities are impaired by osmotic-, ionic-, and oxidative stresses, nutritional imbalance, or a combination of these factors [11]. In fact, plant growth and development are limited by salt stress due to the negative influences through the ionic and osmotic components on various biochemical reactions and physiological processes such as photosynthesis, antioxidant metabolism, mineral nutrient homeostasis, osmolyte accumulation, and hormonal signaling [12]. Most species, including crops, activate tolerance mechanisms only after exposure to salt stress. Activation of the tolerance program drives plants to acclimatize under the saline condition and involves altered physiological responses, redirection of metabolism, reinforcement of defense and repair, and changes in developmental programs to adapt morphological and anatomical characteristics [1,13]. Complex coordination of several signaling pathways is needed to activate the plant responses to salt stress. Phytohormone-mediated signaling, for instance, is crucial in the induction of gene networks related to salt tolerance [14].

Among abiotic stress-related hormones, abscisic acid (ABA) is well known to be the key phytohormone in endogenous signaling that allows plants to survive adverse environmental conditions [15]. The role of ABA for salinity adaptation has long been intensively studied and documented [16,17,18]. More recently, the biological relevance of jasmonate (JA) and its derivatives in the induction of tolerance to abiotic stresses has been demonstrated [19,20,21]. JAs are critical signaling molecules in various development and defense processes of plants [22,23] and play essential roles in plant response to salt stress [24,25,26]. Similar to ABA, accumulation of JAs has been reported in salt-tolerant crops compared to sensitive cultivars [27]. Salt stress has been observed to cause increased levels of JA in leaves and roots and the induction of JA biosynthesis-related genes [28,29,30]. In addition, exogenous application of JA significantly reduces the Na^+^ ion content in salinity-tolerant rice and wheat [27,31] and recovers salt-induced defects in seedling development and photosynthetic activity [32,33]. All this evidence is highly correlated with a positive role of JA in the plant response to salt stress. JA-crosstalk with other phytohormones has aroused the interest of researchers since the interaction among multiple plant hormone signaling integrates environmental and development cues. In this sense, JA has been proposed as a core signal in the phytohormone signaling network [34] because it regulates the balance between plant growth and defense [35,36]. The JA signaling components JASMONATE ZIM-DOMAIN (JAZ) and MYC2 have been identified as the main nodes in the orchestration of JA interplay with other hormone signaling pathways [37,38]. However, the cross-coordination between JA and the other phytohormone is far from being completely understood.

Currently, many strategies have been implemented to obtain tolerant plants to salt stress including classical plant breeding and genetic engineering approaches [39]. However, the complexity of the salt tolerance trait requires more in-depth studies to be understood. Exploring germplasm that possesses genetic variability across a wide spectrum of salt tolerance-related traits could provide valuable information. The study of biparental mapping populations and diversity panels has allowed the discovery of beneficial genetic variants (or alleles) that can be examined for traits that have significant components of salt stress tolerance and their associated quantitative trait loci (QTL) [40]. The analysis of these traits by phenotyping and genotyping techniques could reveal new insights into the biological mechanisms underlying the salt tolerance phenotypes [41]. A widely employed approach to identify the association between each genotyped marker and a phenotype of interest that has been scored across several individuals is the genome-wide association study (GWAS) [42]. It offers some advantages such as more accurate positioning and mapping, simultaneous assessments of multiple alleles at a locus, no requirement for linkage group construction [43,44] and can serve as a basic experiment to identify candidates for mutagenesis and transgenics [45].

With the possibility of performance-specific and predictable genetic modifications through genome (or gene) editing (GE) tools, the information obtained by GWASs becomes more valuable for plant breeding and crop improvement efforts. Among GE methods, the CRISPR-Cas9 system has been used to enhance salt tolerance in some crops like rice and tomato [46,47]. Given that JA regulates processes associated with growth, development, and salt response, the components of JA-biosynthesis, metabolism, and signaling can be targets for phytohormone engineering to produce salt-resilient crops with high yields. Furthermore, increased knowledge of the signals and molecular mechanisms involved in the plant salinity response would pave the path to obtain salt-tolerant crops without productivity penalties.

In this review, we update the information of the role of JA in the salt stress response, mainly in the topics related to JA-crosstalk with other phytohormones in salinity conditions, although limited information about JA-crosstalk during salt stress is available, here we bring together the latest studies on this topic. Finally, we reviewed the use of genome-associated tools to improve salt tolerance by manipulation of JA signaling.

## 2. Jasmonate Metabolism, Signaling, and Response during Salt Stress

### 2.1. JA Biosynthesis, Signaling, and Catabolism

The JA hormones have traditionally been studied in several plant species in the regulation of aspects regarding development, metabolism, and adaptation to biotic stress. However, JAs have lastly been associated with plant responses to several abiotic stresses, adding to JAs extra functions in plant adaptation [20,48]. The JA pathway consists mainly of the JA-associated biosynthesis, signaling, catabolism, and response. According to the information obtained in *Arabidopsis*, JAs are oxylipins, which biosynthesis begins in the plastids. The α-linolenic acid fatty acid, produced by the action of A_1_-type lipases (PLIP and PLA_1_), is transformed into several intermediates by reactions catalyzed by 13-lipoxygenase (13-LOX), allene oxide synthase (AOS), and allene oxide cyclase (AOC), leading to 12-oxo-phytodienoic acid (OPDA) [49,50]. OPDA is translocated to the peroxisome where 12-oxo-phytodienoate reductase 3 (OPR3) and several β-oxidation cycles give rise to jasmonic acid (JA) [51]. The biosynthesis of methyl jasmonate (MeJA) and jasmonoyl-isoleucine (JA-Ile) from JA is catalyzed by jasmonic acid methyltransferase (JMT) [52] and jasmonic acid-amide synthetase 1 (JAR1) [53], respectively. Meanwhile, the reconversion of MeJA and JA-Ile to JA is catalyzed by methyl jasmonate esterase (MJE) [54] and JA-Ile hydrolase 1 (JIH1) [55], respectively.

The JA-Ile molecule is the bioactive JA responsible for the activation of JA responses [56]. The physiological effects mediated by JA-Ile require activation of the JA signaling pathway, which has been well characterized in *Arabidopsis* [57]. The F-box CORONATINE INSENSITIVE1 protein (COI1) is part of the Skp-Cullin-F-box-type E3 ubiquitin ligase complex (SCF^COI1^) and together with JAZ proteins form the JA-Ile receptor [58,59,60,61,62]. In *Arabidopsis*, when JA-Ile level is low, JAZ proteins repress MYC transcriptional activity by recruiting NOVEL INTERACTOR OF JAZ (NINJA) and TOPLESS corepressors [63,64] and by obstructing the association of the coactivator protein mediator complex subunit 25 (MED25) with the transcription initiation complex [65,66,67]. However, once the JA-Ile level rises, it mediates the COI1-JAZ interaction leading to JAZ proteins ubiquitination and degradation by the 26S proteasome. Then, MYC2 and additional transcription factors (TFs) induce the expression of early JA-responsive genes such as JAZs, MYCs, and JA biosynthetic ones [58,68]. MYC2 is a key transcription factor in the JA pathway and acts as a regulatory hub for several biotic and abiotic stress-related responses. Its homologs, MYC3, MYC4, and MYC5, also act alternatively in the JA pathway in *Arabidopsis* [57,69,70]. MYC2 contains a conserved basic helix-loop-helix (bHLH) domain [71], which is required to form homo- or heterodimers with MYC3 and MYC4 [69], although the strongest activation of JA-regulated genes is provided by the MYC2 tetramer [72]. The basic region of MYC2 protein is involved in binding to the sequence 5′-CACGTG-3′, known as G-box, which is present in the MYC2 target promoters [73]. The G-box contains many other variants and weaker binding sequences [74,75]. Furthermore, MYC2 N-terminal contains a putative transcriptional activation domain (TAD) [69], and a JAZ interaction domain (JID) through which interacts with the C-terminal Jas domain of JAZ proteins [58,69]. In several JA-dependent functions, MYC2 regulates many secondary TFs by binding to their promoters, which, in turn, activate downstream gene promoters, creating a hierarchical transcriptional network of JA-mediated response [76,77,78].

Recently, an OPR3-independent pathway for JA synthesis has been described. Chini et al. [79] isolated and characterized the complete knockout mutant of the *opr3-3* allele. Similar to wild-type (WT) plants, the *opr3-3* mutants were resistant to necrotrophic pathogens and insect feeding and activated COI1-dependent JA-mediated gene expression. Through OPDA derivatives analysis the 4,5-didehydro-JA (4,5-ddh-JA) was identified to act as a precursor for JA and JA-Ile biosynthesis in OPR3 absence. The authors demonstrated that in the lack of OPR3, OPDA could enter the peroxisomal β-oxidation pathway to produce 4,5-ddh-JA, which after leaving the peroxisome, is reduced by the cytosolic 12-oxo-phytodienoate reductase 2 (OPR2). This pathway takes place naturally in WT plants and is maximized in the *opr3* mutant [79].

Together with JA biosynthetic and signaling genes, the JA-Ile catabolic genes are strongly coregulated indicating the importance to maintain JA homeostasis [80]. There are two JA-catabolic pathways: one is defined by CYP94B3/B1 and CYP94C1, members of the cytochrome P450 enzymes of the subfamily 94 (CYP94) [81,82,83], and the other one involves the IAA-alanine resistant 3 (IAR3) and IAA-amino acid hydrolase ILR1-like 6 (ILL6) enzymes, members of the amidohydrolase (AH) family [84]. CYP94B3 and CYP94B1 are JA-Ile ω-hydroxylases that generate 12OH-JA-Ile, and CYP94C1 catalyzes a more complete JA-Ile oxidation to 12COOH-JA-Ile [82]. Meanwhile, IAR3 and ILL6 prompt JA-Ile to deactivate by deconjugation reactions [55]. All these enzymes diminish specifically JA-Ile hormone pools to weaken JA signaling as many loss and gain-of-function experiments have demonstrated [85]. The proper regulation and termination of JA-mediated processes are essential to avoid the harmful metabolic effects of a JA amplified response trigger by biotic and abiotic stress. However, it is necessary to highlight that lately, a role of 12OH-JA-Ile in JA signaling has been described [86,87]. Exogenous application of 12OH-JA-Ile mimicked several JA-Ile effects including JA-marker gene expression, anthocyanin accumulation, and trichome induction in *Arabidopsis* [86]. In silico and in vitro assays showed that 12OH-JA-Ile could interact with some COI1-JAZs coreceptors and function as an active jasmonate signal, but more weakly comparing with JA-Ile. It has been proposed that after a strong immune response mediated by JA-Ile, 12OH-JA-Ile modulates JA-Ile activated processes contributing to wound and defense plant response [87].

**Figure 1:**
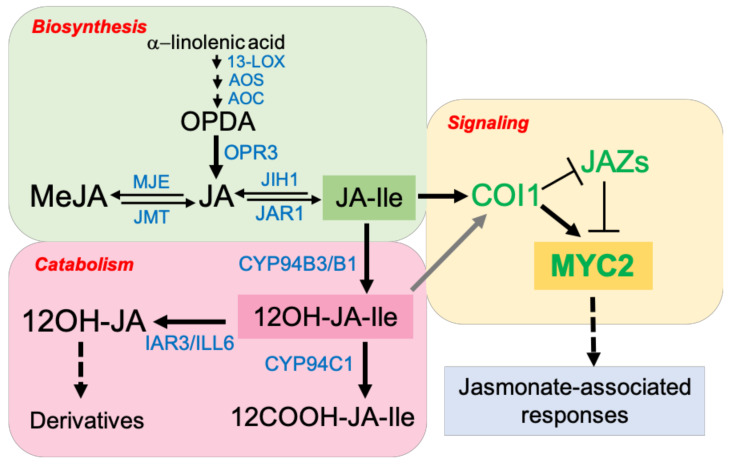
Overview of the jasmonate (JA) pathway including the major molecular players involved in biosynthesis, signaling, and catabolism. Black, blue, and green fonts show the main metabolites, enzymes, and proteins, respectively, for each section of the pathway. Scheme made based on [49,58,80]. For more details, see the text.

In Figure 1, a schematic diagram of the biosynthesis, signaling, and catabolism of JAs is presented.

### 2.2. Salt Stress and JA Response

Earlier reports have shown the induction of some JA biosynthetic genes in *Arabidopsis* roots under salt stress conditions [88,89]. In sweet potato, Zhang et al. [90] studied the root transcriptomes of a salt-sensitive variety and a salt-tolerant line revealing a significant upregulation of the genes involved in the JA biosynthesis and signaling pathways under salt stress. The upregulation in the salt-tolerant line was greater than the sensitive line indicating the essential role of JA in the response of sweet potato to salt stress [90]. The *Arabidopsis* lipoxygenase3 (LOX3) was dramatically induced under salt treatment and the *lox3* mutant exhibited salt hypersensitivity. The *lox3* mutant salt sensitivity phenotype was rescued by the MeJA application indicating the association between JA and salt tolerance [91]. The *TaAOC1* gene from bread wheat responds to salinity and its constitutive expression in both bread wheat and *Arabidopsis* enhanced their level of tolerance to salt stress [92]. On the contrary, the impaired function of AOC in the OPDA-deficient rice ALLENE OXIDE CYCLASE mutants (*cpm2* and *hebiba*) conferred salt tolerance [93]. It is not clear what causes this tolerance, the lack of JA or JA-Ile, or the absence of their precursor 12-OPDA [19].

Several studies in *Arabidopsis* have demonstrated the essential role of MYC2 in salt response mediated by JA signaling [94,95]. The salt and ABA inducible gene *responsive to dehydration 22* (*RD22*) is regulated by MYC2. The RD22 promoter region contains the MYC and MYB recognition sites [96] and the AtMYC2 and AtMYB2 TFs specifically interact with them, respectively. The *atmyc*2 mutant and MYC2 overexpressing (OE) plants treated with ABA showed different *RD22* expression levels, the latter increased the *RD22* expression at low ABA concentration (500 nM) while the former increased *RD22* expression at higher ABA concentration (1 µM) [94]. MYC2 also has an important role in the activation of JA signaling by salt stress on the inhibition of cell elongation in *Arabidopsis* primary roots [95]. Additionally, the salt stress-mediated activation of MYC2 by the MAPK cascade regulates the proline biosynthesis through the delta1-pyrroline-5-carboxylate synthase1 (*P5CS1*) gene, which is a rate-limiting enzyme in the proline biosynthesis pathway [97]. Moreover, Seo et al. [98] demonstrated that the E3 ubiquitin ligase PLANT U-BOX PROTEIN 10 (PUB10), which regulates MYC2 stability [99], positively regulates salt and osmotic stress tolerance during seed germination. It was suggested that PUB10 acts as a negative regulator of ABA signaling through MYC2 and participates in the fine-tuning of ABA signaling and JA crosstalk in the abiotic stress tolerance in plants [98].

In turn, COI1, a core component of the JA-Ile coreceptor [59], is essential for JAZ transcript upregulation in the roots during the response to salt stress [95]. The JAZ upregulation mediated by salt stress in a COI1-dependent manner observed in the roots is likely to follow the canonical JA signaling pathway [100], with proteasome-mediated degradation of JAZ proteins. In this sense, JAZ proteins, the negative regulators of JA signaling, play an important role in the plant response to salt stress. Several JAZ homologous genes were upregulated by NaCl treatment in cotton, *Arabidopsis* roots, tomato, and wheat [95,101,102,103]. Moreover, the OsJAZ9 overexpression in rice resulted in a higher tolerance to salt stress [104] mainly through regulating the expression of ion transporters for K^+^ homeostasis [105]. Similarly, enhancing the expression of OsJAZ8 transcripts assured better performance of transgenic rice lines under salt stress [106]. Furthermore, the rice nuclear factor, RICE SALT SENSITIVE3 (RSS3), forms a ternary complex with class-C bHLH TFs and JAZ proteins and regulates root cell elongation during adaptation to salinity [107]. Remarkably, JAZ genes from *Glycine soja* (*GsJAZ2*), *Malus domestica* (*MdJAZ2*), *Triticum durum* (*TdTIFY11*a), and *Pohlia nutans* (*PnJAZ1*) introduced in *Arabidopsis*, granted greater tolerance to salinity [103,108,109,110]. The introduction of *GaJAZ1* from *Gossypium arboreum* into *Gossypium hirsutum* (upland cotton) significantly increased salt tolerance in upland cotton compared to the WT strain. GaJAZ1-transgenic and WT plants showed many differentially expressed genes involved in JA signaling and biosynthesis, salt stress, and other hormone pathways. In GaJAZ-OE plants, the expression level of *JAZ1/3/6/8* and *JAZ10* were upregulated without NaCl treatment compared to the WT, but under salt conditions they were first downregulated (after 6 h and 12 h of treatment) and then upregulated again (24 h of treatment), indicating a sophisticated regulation of these genes in GaJAZ1-OE plants. Moreover, *MYC2* was significantly upregulated while JAR1 was downregulated in GaJAZ1-OE plants. JA biosynthesis was also affected in GaJAZ1-OE plants since various JA synthesis-related genes (e.g., *LOX*s, *AOS*, *AOC4*, and *JMT*) changed their expression compared to the WT. Additionally, several salt stress-related genes encoding for a vacuolar-associated protein (*VSR1*), an osmotic protein (*OSM34*), and a plasma membrane ion exchanger (*CHX18*) among others showed significant downregulation resulting in the accumulation of osmolytes that protect the plant from salt stress damage. Furthermore, some phytohormone-related genes besides JA-related genes reprogrammed their expression in GaJAZ1-OE plants compared to the WT including those related with ethylene- (*ACS*, *ERF2*, and *ERF4*); ABA- (*ABR1*, *ABA2*, *CBF4*, and *RD26*); and auxin- (*GH3.6*) pathways. In general, these results suggested that ectopic overexpression of *GaJAZ1* affects JA-related genes to increase salt tolerance in *G. hirsutum* plants albeit it also depends on other factors including hormone-crossing signal and salt-inducible genes [111].

Recently, JA-Ile catabolic genes have also been related to salt stress tolerance. Thus, *OsCYP94C2b* overexpression enhanced the viability of the transgenic rice under saline conditions and delayed the salt stress-induced leaf senescence [112]. Similarly, a higher *CYP94C2b* expression has been observed in some salt-tolerant rice varieties indicating that, at least in part, *CYP94C2b* may account for salt tolerance [113]. Hazman et al. [85] analyzed the accumulation of JAs and catabolic compounds in leaves from salt-exposed and control seedlings. OPDA and JA levels were increased by NaCl (100 mM) at most time evaluated points. Correspondingly, the JA-Ile catabolites 12OH-JA-Ile and 12COOH-JA-Ile were enhanced in response to salt exposure. Then, they explored the rice *CYP94* and *AH* gene families and examined the transcriptional response of a gene subset under salt exposure. Among the evaluated *CYP94* genes, only *OsCYP94C2a* was induced by salt stress while the transcripts of the *AH* genes fluctuated marginally. Apparently, *OsCYP94C2a* is the main player of JA-Ile oxidation upon salt stress in rice [85,112,113].

The apparent incongruity in the JA contribution to salt adaptation due to the positive effects of JA exogenous application (see Section 4), the upregulation of JA biosynthesis, and the induction of negative regulators of JA signaling and JA-Ile catabolism, indicates that timing and control of JA are maybe more important than its presence or absence [19,24]. Thus, detailed studies are required to reveal the underlying mechanism of efficient fine-tuning of jasmonate signaling in salt adaptation. Table 1 summarizes the findings regarding the main molecular components of the JA pathway associated with salt tolerance.

It is necessary to highlight that JA does not work independently in the improvement of plant tolerance to salt stress. Instead, its tightened coordination with other phytohormone signaling pathways allows the expression of multiple genes and flux of various metabolic pathways to adjust plant response to stress severity, specifically, in the appropriate time and tissue [18,34].

## 3. Crosstalk between JA and Other Plant Hormones during Salt Stress

In JA signaling, the proteins MYC, JAZ, and COI1 have been established as the core of the pathway and have been pointed to serve as a link between different hormone signaling [116]. Thus, the JA crosstalk with other phytohormones involves these components.

### 3.1. JA and ABA

Several studies have reported the combined action of JA and ABA in the plant response to salt stress. Although an earlier study described that ABA and JA antagonistically regulate the expression of transcripts inducible by salt stress in rice (*Oryza sativa*) [28], in more recent studies the synergistic action of both phytohormones has been observed. For instance, the application of ABA together with different concentrations of JA activated the protection mechanism against NaCl-associated stress in strawberry plants (*Fragaria × ananassa*) [117]. Likewise, ABA and JAs had a synergistic effect on the inhibition of seed germination under salinity conditions [110]. Moreover, Yang et al. [118] confirmed the cooperation between JA and ABA in the tolerance to salt stress mediated by phytochromes. Albeit the evidence of the concerted action of JA-ABA, the cross-coordination that exists between their signaling pathways is just beginning to be understood [119].

MYC2 seems to be an essential point for JA-ABA crosstalk as demonstrated in several studies. Accordingly, *MYC2*-OE *Arabidopsis* plants and the *myc2* mutant show boosted and decreased ABA sensitivity, respectively [94,120]. Additionally, the expression of the salt- and ABA-responsive gene *RD22* is promoted by MYC2 [94,96]. MYC2 and ABI5 (a transcription factor activated by the ABA signaling pathway) are modulated at the protein level through MED25, which is a multifunctional subunit of the *Arabidopsis* mediator complex [121]. In turn, the ABA receptor PYL6 directly interacts and alters the transcriptional activity of MYC2 [122]. Meanwhile, PYL4 is involved in the coregulatory effects of ABA and JA on plant growth and metabolism [123]. Furthermore, the induction of *MYC2* by ABA seems to depend on the JA-Ile COI1 receptor according to Lorenzo et al. [120]. Recently, it has been reported that the application of ABA to strawberry plantlets involves the upregulation of the *MYC2* gene (*FabHLH80*) from 0.5 to 12 h post-treatment [124].

Some studies highlight the importance of JAZ proteins as key nodes of JA-ABA crosstalk. The ubiquitin ligase E3 KEEP ON GOING (KEG), a known ABI5 repressor in the ABA signaling pathway, directly interacts with JAZ12 and modulates its stability [125]. Furthermore, JAZ3 interacts with ABI5 in vivo and represses its transcriptional activity [126]. The overexpression of *PnJAZ1* (isolated from the moss *P. nutans*) in *Arabidopsis* plants inhibited the expression of genes of the ABA-dependent pathway related to seed germination and shoot growth under high salt conditions [110]. Furthermore, the transcription factor GbWRKY1 (from *Gossypium barbadense*) negatively regulated ABA signaling through an interaction network involving JAZ1 and ABI1 (the negative regulator of ABA signaling), in the response to salt and drought stress [115]. The evidence demonstrates the role of JAZs in the response to salt stress and JA-ABA crosstalk, however, the mechanisms of action in which these proteins participate in salt tolerance remain to be fully elucidated.

### 3.2. JA and Other Phytohormones

The nature of jasmonate crosstalk with other phytohormones in salt stress cannot be clearly described due to limited experimental data. At the molecular level, little is known about the convergence points of JA signaling and other phytohormone pathways. Due to the well-established central role of ABA under abiotic stress, more evidence of JA-ABA crosstalk is available [19]. In recent years, the crosstalk among different phytohormone mediating salt stress responses has been described [18]. However, it is necessary to deepen the JA crosstalk with other phytohormones to regulate salt stress tolerance in plants.

#### 3.2.1. JA and Ethylene (ET)

JA–ET crosstalk can be complex and depends on the specific situation. A synergistic effect has been observed in promoting leaf senescence [127,128], but antagonistic interaction was found in controlling apical hook curvature [129]. JA–ET interactions in response to pathogen infections, herbivore attacks, and environmental stress are context-specific. The ET-stabilized transcription factor ETHYLENE-INSENSITIVE3 (EIN3) physically interacts with MYC2 and inhibits its DNA binding activity attenuating JA-regulated plant defense against generalist herbivores [129]. Since the *jaz* decuple mutant showed robust activation of insect and fungal pathogen defenses, the JA–ET crosstalk seems to be mediated via EIN3/ETHYLENE INSENSITIVE 3-like 1 (EIL1) along with JAZs-MYC2 [35]. The two TFs ETHYLENE RESPONSE FACTOR1 (ERF1) and OCTADECANOID-RESPONSIVE ARABIDOPSIS AP2/ERF 59 (ORA59) were previously implicated in integrating JA and ET signaling in *Arabidopsis* [130,131]. Recently, a new convergence-point of JA–ET signaling has been reported. *OsLOX9*, a JA biosynthetic pathway-related gene, is regulated by OsEIL1 in response to piercing-sucking insect attacks [132].

In salt stress, the synergistic activation of the *Arabidopsis* ERF1 by JA and ET is required for inducing tolerance [133]. Moreover, a rice root-specific pathogenesis-related protein (RSOsPR10), induced by high salt and other abiotic stresses, promotes root growth and root mass increasing salt tolerance [134]. JA and ET also induce RSOsPR10, while salicylic acid (SA) almost completely suppresses its induction [135,136]. JA-inducible and OsERF87-dependent expression of *RSOsPR10* were strongly repressed by the SA-inducible OsWRKY76 transcription factor [137]. As OsERF87 and OsWRKY76 bind at the *RSOsPR10* promoter, they antagonistically regulate *RSOsPR10* expression. This mechanism represents a fine-tuning balance between JA/ET and SA signaling in plants under environmental challenges. The description of the molecular components of the synergistic JA–ET crosstalk in the regulation of *RSOsPR10* expression requires investigation. Another gene activated by salt stress and treatment with MeJA or ethephon (an ethylene releasing compound) is *GmCYP82A3*, a gene from the soybean CYP82 family. Transgenic *Nicotiana benthamiana* plants overexpressing *GmCYP82A3* exhibited strong pathogen resistance and enhanced salinity tolerance. Besides, an increased expression of the JA/ET signaling pathway-related genes was observed in the transgenic plants [138]. How GmCYP82A3 is involved in JA–ET crosstalk under salt stress could be an interesting topic to be approached.

#### 3.2.2. JA and SA

The JA–SA antagonism is well known in plant defense pathways and key components of JA–SA crosstalk have been identified. Recently, many of the molecular components in the JA–SA crosstalk that regulate the plant immune network at transcriptional and protein levels are reviewed by Aerts et al. [139]. Among them, MAPKs are involved in the convergence of these phytohormone pathways [140,141,142]. AtMPK4 negatively regulates the activation of SA- and the repression of JA-mediated defenses under biotic stress [140]. Besides, MPK4 positively regulates the glutaredoxin GRX480 in the SA signaling pathway and negatively regulates MYC2 in the JA signaling pathway, which is necessary for JA responsive genes (*PDF1.2* and *THI2.1*) [100]. SA-induced NON EXPRESSOR OF PR GENE (NPR1) activates GRX480, which can block the JA response gene expression mediated by TGA (a D group of *Arabidopsis* bZIP TFs), confirming SA–JA antagonism [143]. Additionally, SA-induced protein kinase (SIPK) and wound-induced protein kinase (WIPK), which are rapidly activated after perception of herbivory, regulate herbivory-induced JA levels and JA-mediated defense metabolite accumulations. WIPK-signaling is associated with large fitness costs in competing *Nicotiana attenuata* plants, while SIPK acts as an important regulator of plant fitness, possibly modulating SA-JA crosstalk through ethylene signaling [142].

The role of JA and SA in salt stress tolerance has been explored previously [144], but their relationship under salinity is not well known yet. Several reports show that SA and JA can alleviate the hazardous effect of salt stress on plants. In strawberry, improved physiological characters such as increased antioxidant activity and a reduced Na^+^/K^+^ ratio were observed on MeJA and SA treatments [145]. Likewise, soybean performance under salinity was improved by foliar spraying of JA and SA [146]. Treatment of soybean plants with SA plus JA stimulated H^+^-ATPase activity of tonoplast, nutrient uptake, and salt tolerance [147]. Additionally, exogenous applications of JA and SA decreased the concentration of Na^+^ in soybean under different salt stress levels [148]. Moreover, MeJA treatment can protect plants from salt-induced damage by acting role as an antioxidant and cooperating with SA [149]. All the evidence invites a deepening in the JA–SA interplay under salinity conditions for identifying the molecular components of their crosstalk.

#### 3.2.3. JA and Gibberellins (GA)

JA and GA signaling pathway interaction has been described that occurs by the key GA signaling proteins DELLAs and JAZs since they directly interact [150]. The JAZ–DELLA interaction would interfere with the inhibition of MYC2 by JAZ proteins resulting in the activation of MYC2 downstream genes. In presence of GA, DELLA proteins are degraded and JAZs bind MYC2 to inhibit JA signaling [150,151]. On the other hand, the *della* mutant is less sensitive to the plant growth inhibition mediated by JA suggesting that JA delays the degradation of DELLA mediated by GA [152]. Curiously, JA signaling upregulates the transcription of *RGL3*, a DELLA gene, which the promotor is a target of MYC2 [153]. Additionally, RGL3 physically interacts with JAZ1 and JAZ8 [64,153] suggesting the JA-mediated degradation of JAZ1 and consequent release of MYC2 to induce the *RGL3* expression, which in turn binds the non-JA degradable JAZ8 enhancing the MYC2-dependent JA responses [153]. Recently, it was reported that JA and GA synergistically promote fiber cell initiation of cotton (*G. hirsutum*) possibly mediated by the GhJAZ3 and GhSLR1 (a DELLA protein) interaction [154].

The interplay of JA and GA under salt conditions has not been studied in depth. However, there is evidence that in salt-stressed plants of basil (*Ocimum basilicum*) the GA concentration significantly decreased and in non-stressed plants treated with JA. Stressed plants treated with JA also showed a significant decrease in GA concentration [155] showing the antagonistic effect of JA in the GA level under salt stress. The effects of the combination of MeJA and NaCl treatment on the growth regulation and defense response of *Nitraria tangutorum*, a desert halophyte, have been recently investigated [156]. Compared with NaCl treatment alone, MeJA treatment aggravated the growth inhibition of seedlings by antagonizing growth-related hormones like GA. It was demonstrated that the transcript levels of GA-responsive genes *NtPIF3*, *NtGAST1,* and *NtGSAT4* were suppressed by MeJA [156]. More studies are needed to find out the components of JA–GA crosstalk under saline conditions.

#### 3.2.4. JA and Cytokinin (CK)

Interactions between JA and CK could grant some developmental flexibility under stress conditions since they mediate stress response and developmental processes. Although there are scarce reports on JA–CK crosstalk, some data indicate that their interaction could be negative or positive [157,158]. Antagonistic effects of JA and CK have been observed in different processes such as senescence, photosynthesis, RNA and protein synthesis, and vascular formation [157,159,160,161]. In contrast, a positive interplay between these phytohormones in delaying senescence of the *Iris* flower (*Iris × hollandica*) has been reported [158]. Moreover, CK treatments promoted gene expression of JA-amino synthetase in *Arabidopsis* and tomato plants [162] suggesting an interaction between the JA–CK pathway.

Recently, Avalbaev et al. [163] demonstrated the influence of exogenous MeJA on endogenous CK content in wheat plants under normal and salinity conditions. Low concentrations of MeJA (0.01–1 µM) increased wheat seedling growth while higher concentrations (10 and 100 µM) inhibited it. The hormonal balance of wheat seedlings was shifted by exogenous application of 0.1 µM MeJA. In response to salt stress, MeJA-untreated wheat plants increased the ABA level and gradually decreased indole acetic acid (IAA) and CK contents. Meanwhile, MeJA-pretreated seedlings were characterized by a diminution of ABA accumulation and IAA decreased level induced by salinity. Noticeably, a salinity-induced decline in the CK content was completely preventive by MeJA, which eventually resulted in the maintenance of the wheat seedlings growth rate under salt stress. It was suggested that MeJA application influences CK metabolism since cytokinin oxidase (CKX) gene expression and enzyme activity decreased after 1 h of MeJA treatment [163]. CKX catalyzes the degradation of CKs and controls the CK content in plants [164]. Similar results were obtained on almond rootstocks when MeJA application (0.025–0.05 mM) increased CK concentration in the leaf due to restriction of CKX activity and its gene expression [165]. MeJA-induced protection against salinity was found to be reached by modulating the activity of the antioxidant system and accumulation of osmoprotectants [165,166]. However, the mechanism by which exogenous MeJA mitigates the effect on the growth of salt-stressed plants as a result of the inhibition of CK decline under salt stress is largely unknown.

#### 3.2.5. JA and Auxin (AUX)

The JA–AUX crosstalk was early reported by the identification of the *auxin-resistant1* (*axr1*) mutants with altered jasmonate responsive gene expression [167]. Moreover, a link between JA signaling and AUX homeostasis was evidenced through the JA-mediated modulation of *YUCCA8* and *YUCCA9* gene expression, which are involved in AUX biosynthesis [168]. JA and AUX interaction also involves the ETHYLENE RESPONSE FACTOR109 (ERF109) during JA-induced lateral root formation [169]. Recently, Xu et al. [170] reviewed the novel progresses on the integration of JA and ethylene into AUX signaling in regulating root development of *Arabidopsis thaliana* [170]. Besides, Zhang et al. [171] provided an example of metabolic-level crosstalk between the JA and AUX signaling pathways by demonstrating that wounded leaves JA-inducible amidohydrolases (ILR1, ILL6, and IAR3) contribute to regulate active IAA and JA-Ile levels, promoting AUX signaling while attenuating JA signaling [171].

The transcription factor WRKY57, which is upregulated by IAA, but downregulated by JA, has been described as a convergence node of JA and IAA-mediated signaling. The JAZ4/8 and IAA29 (an AUX/IAA protein) repressors of JA and IAA signaling, respectively, have an opposite function in WRKY57 regulation since both competitively bind WRKY57 during JA-induced leaf senescence in *Arabidopsis* [172]. In earlier work, Jiang et al. [173] found that the *Arabidopsis adt* mutant, which is constitutive to expressing the *WRKY57* gene, exhibited drought, osmotic, and salt tolerance. The enhanced tolerance of the *adt* mutant to these stresses was associated with an increment of ABA content consistently with the upregulation of *RD29A*, *NCED3*, and *ABA3* genes. It was demonstrated that WRKY57 could directly bind the W-box of *RD29A* and *NCED3* promoter sequences, suggesting that WRKY57 could regulate their expression. Since WRKY57 is regulated by JAZ4/8 and IAA29 repressors [172], it could be interesting to evaluate their role in the plant response to salt stress. Perhaps, it would shed light on the JA–AUX interplay under salinity conditions.

The main molecular/physiological effects related to phytohormone crosstalk are summarized in Table 2.

## 4. Effects of JA-Exogenous Applications for Improving Salt Stress Tolerance

Exogenous jasmonate applications have effects on different physiological aspects, including protection against biotic and abiotic stresses [174,175,176]. Methyl jasmonate (MeJA) application induces protection against oxidative stress as has been reported in different species and conditions [176,177,178,179].

In *Arabidopsis thaliana*, the total activities of catalase (CAT), peroxidase (POD/POX), superoxide dismutase (SOD), and glutathione reductase (GR) increased considerably in response to MeJA [180]. In crops, such as strawberry, MeJA applications during the preharvest period increase the anthocyanin and ascorbic acid contents, CAT, and ascorbate peroxidase (APX) activities in fruits at postharvest storage [176]. In this sense, increased activity of the antioxidant enzymes, together with higher levels of antioxidant compounds, as a result of MeJA treatment reinforces the antioxidant response of plants to reactive oxidative species (ROS) caused by abiotic stresses, like high salt.

From a physiological point of view, salt stress increases free proline content, photorespiration, and stomatal resistance among others, while decreasing net photosynthetic rates, transpiration, protein, and relative water content (RWC) [181,182]. Pretreatment with 0.1 mM MeJA helps the pea seedlings to counteract the salt stress since RWC and protein content of the treated seedlings were higher in comparison to NaCl-treated seedlings. Moreover, MeJA-treated pea seedlings present a decrease of Na^+^ and Cl^−^ accumulation in the shoot [183]. In another experiment, pretreatment with 0.1 mM MeJA for 3 days before salt treatment diminished the inhibitory effect of NaCl on the rate of ^14^CO_2_ fixation, and activity and content of ribulose-1,5-bisphosphate carboxylase/oxygenase [184]. Additionally, in rapeseed (*Brassica napus*), exogenously applied MeJA counteracted the inhibitory effects of NaCl by increasing RWC, soluble sugar content, and photosynthesis rate [174]. The application of 0.25 mM MeJA was the most effective treatment to enhance salt tolerance at a concentration of 60 mM NaCl in strawberry (*F.* × *ananassa* ‘Camarosa’) seedlings [145]. In almond rootstocks, application of MeJA in optimal concentrations of 0.025–0.05 mM alleviated the adverse effect of salt stress by increasing the photosynthetic rate, activity of antioxidant enzymes (APX, SOD, and POX), root and shoot dry mass, and cell membrane integrity [165]. Alleviation of moderate salinity (40 mM NaCl) by foliar application of 5 mM MeJA has also been reported in broccoli [185]. Pretreatment of cowpea seeds with 0.05 mM MeJA improves plant tolerance to salt stress [186].

In other plants, like *Limonium bicolor*, which is a typical recretohalophyte with salt glands in the epidermis, 300 mM NaCl led to a dramatic inhibition of seedling growth that was significantly alleviated by the application of 0.03 mM MeJA, resulting in biomass close to that of plants not subjected to salt stress [187]. Even in high salt concentrations such as 500 mM NaCl, MeJA applied at 0.1 mM has a protective role in the defense response of *Robinia pseudoacacia* especially with a marked increase in the activity of antioxidant enzymes and related gene expression [188]. In another study, foliar applications of 0.5 mM MeJA increased the essential oil content and the antioxidant activities of basil (*O. basilicum* ‘Genove’) on 30 mM NaCl and have noticeable effects on the main components of the oils [189]. In *Glycyrrhiza uralensis* exposed to 100 mM NaCl, 0.025 or 0.05 mM MeJA increased the root length of salt-stressed *G. uralensis* seedlings but decreased root diameter, stem length, and stem diameter, enhancing peroxidase activity and ascorbate content [190].

In wheat seedlings, NaCl salt stress caused a significant increase in the malondialdehyde (MDA) content and H_2_O_2_ concentrations and a concomitant decrease in SOD, POD, CAT, and APX activities. Exogenous JA pretreatment (2 mM) combined with NaCl treatment (150 mM) produced a significant decline in MDA and H_2_O_2_ concentrations and an increase in SOD, POD, CAT, and APX activities. Moreover, a marked upregulation of SOD, POD, CAT, and APX genes was observed in the JA–NaCl combined treatment in comparison to NaCl treatment alone. Additionally, exogenous JA remarkably increased glutathione (GSH) concentration in wheat seedlings treated with NaCl and decreased the deleterious effect of salt stress on the growth of wheat [31]. These results indicate that exogenous JA can effectively scavenge ROS by enhancing the activities of antioxidant enzymes and the concentration of antioxidant compounds in wheat seedlings under salt stress and consequently play an important role in decreasing lipid peroxidation and increase the ability of wheat to resist salt stress [31].

In roselle (*Hibiscus sabdariffa*), the exogenous JA treatment protected roselle seedlings against salt-induced harms through enhancing the activities of both enzymatic and non-enzymatic antioxidants, such as APX, pyrogallol peroxidase (PPX), and PPO, and the accumulation of metabolites non-reducing sugars, total phenols, anthocyanins, flavonoids, and proline. The JA-treated roselle exhibited a significant increase in growth parameters under salt conditions compared to the WT [191]. In forage sorghum (*Sorghum bicolor*), at a high salinity level (200 mM), seeds treated with 10 mM JA showed a positive effect on various growth and physiological parameters such as emergence percentage, emergence rate, shoot length, total fresh weight, salt tolerance index, and total chlorophyll among others [192]. Similarly, seed priming and foliar application with JA enhanced salinity stress tolerance of soybean (*Glycine max*) seedlings. Improved water and osmotic potentials, water use efficiency, net photosynthetic, transpiration rate, stomatal conductance, and total chlorophyll content were observed in JA-primed and treated soybean seedlings compared to the untreated ones. Besides, JA treatment resulted in a reduction of Na^+^ concentration and an increment of K^+^ concentrations in the leaf and root of the analyzed cultivars despite salinity stress [193].

Although the exogenous application of JA and its derivates constitute a suitable approach to improve the plant response to salt stress, this strategy has some limitations. Due to the JA–SA antagonism during the plant defense response [194], an increment in JA content by its exogenous application will negatively affect the SA-mediated response to biotrophic pathogens. In addition, JA can inhibit plant growth by reprogramming plant metabolism to produce diverse defense compounds [35], thus the growth-defense trade-off is also an issue. Moreover, the high economic cost of MeJA application in field treatments [176] could prevent its use to mitigate the salt-induced damage in crop plants. It is necessary to keep in mind that the activation of JA-beneficial effects in plant response to salt stress depends on the JA levels and therefore, field experiments will be required to analyze the cost-effective JA doses for exogenous applications.

## 5. Application of Genome-Associated Tools for Salt Tolerance Mediated by JA

Salt stress tolerance is a complex trait regulated by polygenes [195]. In this context, QTL mapping and GWASs provide a suitable opportunity to identify genes responsible for quantitative trait variation such as salt tolerance. In this regard, genetic factors associated with salt stress have been previously investigated in several crops, such as rice [41,196,197], barley [198,199], wheat [200,201], chickpea [202], sesame [203], cotton [204,205], and soybean [206].

**Figure 2:**
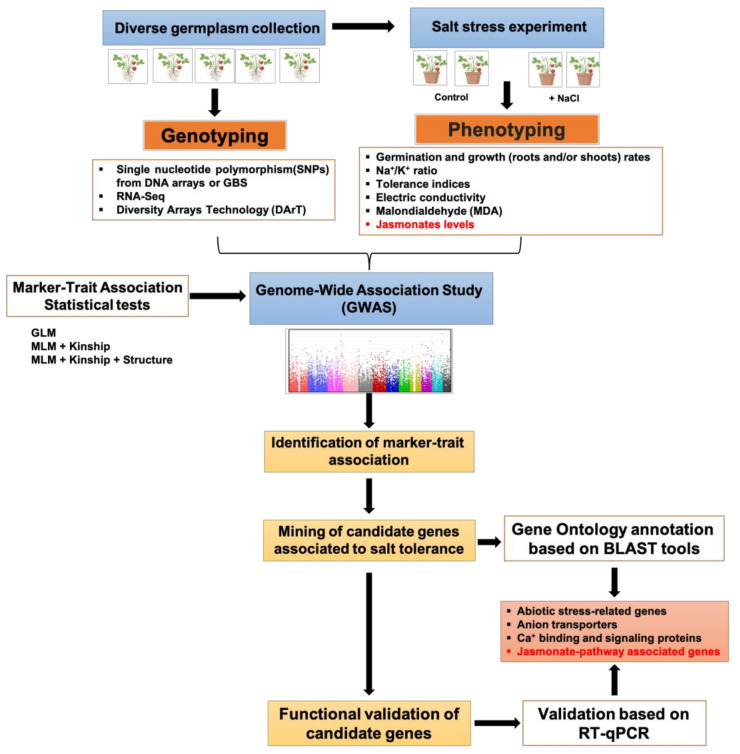
Genome-wide association study (GWAS) pipeline to identify jasmonate pathway-associated genes involved in the salt stress response and salt tolerance in crops. GBS, SSI, GLM, and MLM correspond to genotyping by sequencing, salt stress index, general linear model, and mixed linear model, respectively. For more details, see the text.

GWAS and QTL mapping have been implemented to identify the genetic factors involved in both osmotic and ionic components of salinity stress. A typical GWAS workflow to identify genes related to salt stress (and genes conferring salt tolerance) in crops is represented in Figure 2. Four main steps can be distinguished: (1) plant genotyping; (2) plant phenotyping based on morphological and physiological traits related to salt stress; (3) identification of marker–trait associations (MTAs); and (4) identification of candidate genes involved in the salt stress response. A diverse panel is genotyped using DNA markers, i.e., single nucleotide polymorphisms (SNPs), diversity arrays technology (DArT), or RNA-sequencing (RNA-seq) for the identification of genetic variants that affect the gene expression level in a specific tissue. Parallelly, this panel is phenotyped for different traits regarding the objectives of the study. In the context of salt stress in plants, the phenotyping is generally carried out considering several morphological and physiological traits, such as leaf area, root and shoot dry weight, seed germination rate, salt stress index (SSI), Na^+^/K^+^ ratio, chlorophyll content, and MDA, and, remarkably, changes in the phytohormone levels such as JAs. Particularly, these traits are evaluated in plants or seedlings that have been subjected to salt treatment. Subsequently, the genotypic and phenotypic data are combined to associate alleles with particular traits using classical GWAS models, which significantly detect molecular markers associated with the studied traits. Posteriorly, the MTAs can serve as a starting point for the mining of candidate genes. Gene Ontology (GO) annotation of the putative candidate genes can be carried out using BLAST tools.

In a GWAS for salt tolerance (or salt stress), the candidate genes are usually associated with gene annotations, such as abiotic stress-related, anion transport, Ca^+^ binding and signaling, phytohormone response elements, and others [207,208,209]. In this way, many TFs and downstream genes related to phytohormones biosynthesis and signaling have been characterized, including ABA, SA, JA, ET, and others considered as growth promotion hormones, including AUX, GA, and brassinosteroids [41,205,209,210,211,212,213]. Additionally, the candidate genes associated with salt stress or tolerance can be validated by quantitative reverse transcription PCR (RT-qPCR) analysis.

To our knowledge, several studies have analyzed the JA response to different stresses, but few have explored JA-dependent genetic mechanisms taking into account natural genetic variation. To et al. [214] performed a GWAS to identify genetic variants associated with exogenous JA treatment responses in rice. They found a high natural variability for the shoot and root growth (in a panel of 150 rice accessions) in response to JA treatment. This GWAS revealed about 230 candidate genes, including several JA-responsive TFs known to play a stress response role. Several GWASs have elucidated the participation of JA in salt tolerance in plants. In this sense, Li et al. [203] reported potential candidate genes related to drought and salt-induced stress in sesame. In this GWAS, the *SiOPR3* gene (detected for drought stress in sesame) is the ortholog of the *Arabidopsis OPR3* gene an essential component of the JA biosynthesis.

Besides, Rohila et al. [41] detected several candidate genes associated with seedling stage salt tolerance by the GWAS approach in a rice core-collection. Particularly, one SNP (on chromosome 3) was located close to the *ALLENE OXIDE CYCLASE 1* (*AOC1*) gene. This gene has been related to salt and other abiotic stresses, and it is a key gene in the JA biosynthetic pathway in *Arabidopsis* [215]. Yuan et al. [205] combined the association mapping and RNA-seq analyzes to explore candidate genes for salt-tolerance in cotton at the germination stage. At least nine genes were associated with signaling or a response to signal factors, such as SA, GA, and JA. In other GWASs, genes related to the JA biosynthesis and signaling pathways have been indirectly identified, in which upstream genes from the JA signaling events and JA-regulated genes during salt stress were detected. For instance, An et al. [216] carried out a GWAS to identify associations conferring salt tolerance in rice. In this study, a significant SNP for seedling length was located on the promoter of a salt stress-related gene (*RSOsPR10*), which has been proposed to be induced by biotic and abiotic stresses, via the JA signaling pathway [135]. Sun et al. [204] reported DNA polymorphisms associated with salt tolerance candidate genes at the cotton seedling stage. Additionally, the expression levels of six genes were reported using salt-tolerant and salt-sensitive varieties. Interestingly, the expression of *Gh_A10G1756*, a homolog of the *Arabidopsis CALCIUM-DEPENDENT PROTEIN KINASE 1* (*AtCPK1*) gene, had higher expression in the salt-sensitive than salt-tolerant types [204]. The *AtCPK1* gene mediates pathogen resistance in *Arabidopsis* and plays a positive role in salt/drought-stress response [217]. According to Coca and San Segundo [218], *AtCPK1*-OE plants showed activation of two components of the chloroplastic pathway for JA biosynthesis (*AOS* and *AOC* genes), which could support the idea that JA is a key element for triggering different responses to salt stress in plants. Patishtan et al. [208] implemented a GWAS using a diversity panel of 306 rice accessions treated with different salt concentrations. The authors characterized more than 30 candidate genes for short, medium, and long-term NaCl treatment. Notably, among these genes, a DNA polymorphism was associated with the *WRKY70* gene, which plays a pivotal role in an antagonistic interaction between SA and JA responses [210,219].

Due to the above-mentioned studies, GWAS and association studies have successfully identified many novel genes associated with traits of interest. These findings will be useful towards the developing of varieties tolerant to salt stress, using new genomic techniques, such GE; an aspect that has not been addressed in depth. In this sense, GE systems provide the ability to modify genes and generate new possibilities for crop improvement precisely. The RNA-guided CRISPR/Cas9 technology is simple to use across different genome editing technologies [220] and has been generally used in major crops such as wheat [221], maize [222], soybean [223], and many others. Even more, the highly efficient multiplex editing toolkit based on an intron-optimized zCas9i gene, which allows assembly of nuclease constructs expressing up to 32 sgRNAs [224], can enable simultaneously targeting of multiple independent loci to generate complex genotypes or to functionally interrogate groups of candidate genes such as those involved in phytohormones signaling.

To improve the salt tolerance in crops employing the CRISPR/Cas9 system with a focus on phytohormones, an example is the targeting of the *OsRR22* gene, which encodes a transcription factor involved in CK signaling, which has effectively enhanced salt tolerance in rice [46]. Recently, Liu et al. [225] enhanced drought tolerance in tomato (*Solanum lycopersicum*) by CRISPR/Cas9 targeted mutagenesis of the *SlLBD40* gene. *SlLBD40* encodes an organ boundaries domain transcription factor, which is highly induced by polyethylene glycol (PEG), salt, and MeJA treatments. The analysis of the *SlLBD40* expression in the *jasmonic acid-insensitive1* (*jai1*) mutant (a mutant in the JA-Ile tomato receptor COI1) and *MYC2*-silenced plants demonstrated that *SlLBD40* depends on JA signaling for its activation and it might be downstream of *SlMYC2* [225]. The previous works offer a good background for targeting key components of the JA pathway to obtain high salinity tolerance in crops using GE tools.

## 6. Concluding Remarks

Besides the role of development and abiotic stress responses, the jasmonate pathway is certainly involved in plant salt stress responses. Several JA-biosynthetic genes are induced under salt stress, although the lack of jasmonates is also related to this tolerance. Key JA-associated molecular components such as the transcription factor MYC2 and the repressor JAZ seem to be crucial in salt tolerance. Remarkably, overexpression of *JAZ* genes confers salt tolerance in transgenic plants. Regarding phytohormone crosstalk between JA and others in a salt stress context, certainly, more information exists about JA-ABA crosstalk, focused on MYC2 and JAZ interactions with the ABA signaling-components.

In terms of future applied perspectives, on the one hand, several reports demonstrate the positive effects of JA exogenous applications on the physiological status against salt stress. It should be considered as complementary crop management to face not only salinity-derived damages but also all abiotic stresses in a global change framework. On the other hand, genomic tools such as GWAS could help to reveal several JA pathway-associated genes that can serve as a guide in breeding programs and targets in genome editing systems, such as CRISPR/Cas9, to get salt-resistant crops. Surely, the next years will be promising in discoveries and applications related to the role of jasmonates against salt stress in plants.

## Figures and Tables

**Table 1:** Participation of jasmonate (JA) pathway-associated molecular components in salt stress responses of different plants.

Protein	Function	Salt Stress Response	Species	References
Lipoxygenase3 (LOX3)	JA biosynthesis	Induced under salt stress. Methyl jasmonate (MeJA) rescued the salt sensitivity phenotype of the *lox3* mutant	*Arabidopsis thaliana*	[91]
Allene oxide cyclase (AOC)	JA biosynthesis	Constitutive expression enhances tolerance to salt stress	*Triticum aestivum* *A. thaliana*	[92]
		*cpm2* and *hebiba* mutants display salt tolerance	*Oryza sativa*	[93]
MYC2	JA signaling	Transcriptional activator of the salt- and abscisic acid(ABA)-responsive gene *RD22*	*A. thaliana*	[94,96]
		An important role in salt-mediated JA-dependent inhibition of cell elongation in the elongation zone of primary root	*A. thaliana*	[95]
		Its salt stress-mediated activation by MAPK cascade regulates proline biosynthesis	*A. thaliana*	[97]
		Mediates the negative regulation of ABA signaling by PUB10, which acts as a positive regulator for salt and osmotic stress tolerance	*A. thaliana*	[98,99]
Jasmonate ZIM-domain (JAZ)	JA signaling	Induced under salt stress	*A. thaliana* *Gossypium hirsutum* *Solanum lycopersicum*	[95]
		*PnJAZ1* inhibited expression of ABA-dependent genes related to seed germination and shoot growth under high salt conditions	*A. thaliana* *Physcomitrella patens*	[110]
		*OsJAZ9* and *OsJAZ8* overexpression enhanced salt tolerance	*O. sativa*	[104]
		Heterologous expression of *GsJAZ2* and *MdJAZ2* enhanced tolerance to salinity	*A. thaliana*	[114]
		Overexpression of *TdTIFY11a* variants confer salt tolerance to *Arabidopsis* seedlings	*A. thaliana*	[103]
		*GaJAZ1* overexpression significantly increased salt tolerance	*G. hirsutum*	[111]
		*GbWRKY1* overexpression negatively affects salt tolerance through an interaction network involving JAZ1 and ABI1	*A. thaliana*	[115]
Cytochrome P450 family (CYP94C2b)	JA catabolism	*OsCYP94C2b* overexpression enhanced viability under salt conditions and delayed the salt stress-induced leaf senescence	*O. sativa*	[112]

**Table 2:** Molecular and physiological effects of the jasmonate (JA) crosstalk with abscisic acid (ABA), ethylene (ET), salicylic acid (SA), gibberellins (GA), cytokinin (CK), and auxin (AUX) in different plants under salt stress conditions. For more details, see the text.

Crosstalk	Molecular/Physiological Effects	Species	References
JA-ABA	JA and ABA applications in conjunction activate the antioxidant mechanism against salt stress	*Fragaria × ananassa*	[117]
	Synergistic effect on the inhibition of seed germination under salinity conditions	*Arabidopsis thaliana*	[110]
	Synergism in the salt tolerance mediated by phytochrome A and B	*Nicotiana tabacum*	[118]
JA–ET	Synergistic upregulation of *AtERF1* required to induce salt tolerance	*A. thaliana*	[133]
	Synergistic upregulation of *RSOsPR10* which promotes root growth and increases salt tolerance	*Oryza sativa*	[136]
	Synergistic upregulation of *GmCYP82A3* which enhances salinity tolerance	*Glycine max* *Nicotiana benthamiana*	[138]
JA–SA	Methyl jasmonate (MeJA) and SA application increases antioxidant activity and reduced the Na^+^/K^+^ ratio	*F. × ananassa*	[145]
	JA and SA application protects plants from salt-induced damage and improves plant performance under salt conditions	*G. max*	[146]
	JA and SA application stimulates H^+^-ATPase activity of tonoplast, nutrient uptake, and salt tolerance	*G. max*	[147]
JA-GA	JA application decreases GA content in salt-stressed plants	*Ocimum basilicum*	[155]
	MeJA application suppresses the transcript levels of the GA-responsive genes *NtPIF3*, *NtGAST1,* and *NtGSAT4*	*Nitraria tangutorum*	[156]
JA-CK	MeJA application prevents the salinity-induced decline of endogenous CK by reducing the cytokinin oxidase enzymatic activity and its related gene expression	*Triticum aestivum* *Prunus dulcis*	[163]
JA-AUX	An opposite function of JAZ4/8 and IAA29 repressors on the regulation of WRKY57. Constitutive activation of WRKY57 in *adt* mutant confers salt tolerance	*A. thaliana*	[172,173]

## Data Availability

Not applicable.
